# Serum immunoglobulin free light chains and their association with clinical phenotypes, serology and activity in patients with IgG4-related disease

**DOI:** 10.1038/s41598-021-81321-5

**Published:** 2021-01-19

**Authors:** Eduardo Martín-Nares, Vanessa Saavedra-González, Reynerio Fagundo-Sierra, Blanca Estela Santinelli-Núñez, Teresa Romero-Maceda, Karla Calderón-Vasquez, Gabriela Hernandez-Molina

**Affiliations:** 1 Department of Immunology and Rheumatology, Instituto Nacional de Ciencias Médicas y Nutrición Salvador Zubirán, Vasco de Quiroga 15, Col. Belisario Dominguez Sección XVI, 14080 Mexico City, Mexico; 2 Central Laboratory, Instituto Nacional de Ciencias Médicas y Nutrición Salvador Zubirán, Mexico City, Mexico; 3 Clinical Laboratory, Tumor Markers Unit, Instituto Nacional de Cancerología, Mexico City, Mexico

**Keywords:** Autoimmunity, Immunological disorders, Biomarkers, Medical research, Rheumatology

## Abstract

The clinical utility of serum immunoglobulin free light chains (sFLC) in IgG4-related disease (IgG4-RD) is unknown. Herein we evaluated their association with clinical phenotypes, serology and activity in patients with IgG4-RD. Cross-sectional study that included 45 patients with IgG4-RD, and as controls 25 with Sjögren’s syndrome (SS) and 15 with sarcoidosis. IgG4-RD patients were classified in clinical phenotypes: pancreato-hepato-biliary, retroperitoneum/aorta, head/neck-limited and Mikulicz/systemic; as well as proliferative vs. fibrotic phenotypes. We assessed the IgG4-RD Responder Index (IgG4-RD RI) at recruitment and measured IgG1, IgG4, κ and λ sFLC serum levels by turbidometry. sFLC levels were similar among IgG4-RD, SS and sarcoidosis groups. Regarding the IgG4-RD patients, the mean age was 49 years, 24 (53.3%) were men and 55.5% had activity. Eight (17.7%) belonged to pancreato-hepato-biliary, 6 (13.3%) to retroperitoneum/aorta, 14 (31.1%) to head/neck-limited, 16 (35.5%) to Mikulicz/systemic phenotypes, whereas 36 (80%) to proliferative and 9 (20%) to fibrotic phenotypes. High κ sFLC, λ sFLC and κ/λ ratio were present in 29 (64.4%), 13 (28.9%) and 13 (28.9%) of IgG4-RD patients, respectively. There were no differences in sFLC among IgG4-RD phenotypes. κ sFLC and κ/λ ratio correlated positively with the number of involved organs and IgG4-RD RI. Patients with renal involvement had higher κ sFLC and λ sFLC. The AUC for κ sFLC and λ sFLC, for renal involvement was 0.78 and 0.72, respectively. Active IgG4-RD had higher levels of κ sFLC and more frequently a high κ/λ ratio. The AUC for κ sFLC and κ/λ ratio for predicting active IgG4-RD was 0.67 and 0.70, respectively. sFLC correlated positively with IgG1 and IgG4 levels. sFLC may be useful as a biomarker of disease activity as well as multiorgan and renal involvement. In particular, a high κ/λ ratio may identify patients with active disease.

## Introduction

Physiologically during immunoglobulin synthesis more light chains than heavy chains are produced. Consequently, the unbound light chains can be detected in serum as free light chains (sFLC), either κ or λ^1^. During pathological conditions such as monoclonal gammopathies, the measurement of sFLC is clinically useful and the detection of an excess of one light chain over another using the κ/λ ratio is accepted as a surrogate for monoclonality^2^. Thus, assessment of sFLC is of paramount importance in the diagnosis and monitoring of multiple myeloma, AL amyloidosis, or monoclonal gammopathy of undetermined significance^2,3^.

Systemic autoimmune diseases such as Sjögren’s syndrome (SS) or systemic lupus erythematosus (SLE) are accompanied by over activation of B cells and plasma cells, and consequently by polyclonal hypergammaglobulinemia. Thus, the potential utility of measuring sFLC in these disorders has been investigated. For instance, a recent study including 45 patients with SS disclosed that 64% had abnormal sFLC levels and 11% had a high κ/λ ratio; furthermore, sFLC levels correlated with systemic activity and their levels were modified by treatment with rituximab and abatacept, showing a sensitivity to change^4^. Other studies in patients with SLE, rheumatoid arthritis (RA) and systemic sclerosis (SSc) have reported abnormal levels of sFLC and a potential utility as biomarkers of disease activity as well^5–7^.

IgG4-related disease (IgG4-RD) is a recently described immune-mediated condition characterized by tumefactive fibrosclerosing lesions, high serum IgG4 levels and tissue infiltration by IgG4 positive plasma cells, accompanied often by polyclonal hypergammaglobulinemia^8^. Serum IgG4 levels and oligoclonal plasmablasts are the two main biomarkers for diagnosis and/or prognosis accepted to date^9,10^. However, both tools had their limitations: IgG4 levels may be normal in up to a third of patients with IgG4-RD^11,12^ and could be elevated in other conditions that are typically part of the differential diagnosis with IgG4-RD such as pancreatic malignancies, Erdheim-Chester disease and systemic vasculitides^13–16^; whereas, oligoclonal plasmablasts had a better specificity and positive predictive value, but their determination by flow cytometry is not easily accessible. Due to these shortcomings, different biomarkers have been proposed for IgG4-RD such as serum soluble IL-2 receptor, cc-chemokine ligand 18 and B cell-activating factor of the tumor necrosis factor family^17–19^.

The clinical utility of sFLC assessment in IgG4-RD is not known. Thus, in this study we aimed to determine the levels of κ and λ sFLC and the κ/λ ratio in patients with IgG4-RD, their utility to distinguish IgG4-RD from mimickers and their association with clinical phenotypes, serology and activity.

## Results

We included 52 patients with IgG4-RD and 40 controls (25 with SS and 15 with sarcoidosis). Seven patients with IgG4-RD were excluded due to eGFR < 60 mL/min/1.73 m^2^ attributable to kidney and retroperitoneal involvement, age and comorbidities. Forty-five patients with IgG4-RD were included in the analysis with a mean age of 49.4 ± 15.3 years; twenty four (53.3%) were male. Age among IgG4-RD patients and controls was similar (IgG4-RD = 49.4 ± 15.3 vs. SS = 55.3 ± 12.3 vs. sarcoidosis = 43.1 ± 16.6 years, p = 0.08) and SS patients were less frequently male (IgG4-RD = 53.3% vs. SS = 4% vs. sarcoidosis = 33.3%, p < 0.001).

Seven (15.5%) patients had single organ, 7 (15.5%) 2 organs and 31 (68.8%) 3 ≥ organs involved during the course of the disease. Most frequently involved organs were: submandibular glands in 26 (57.8%), pancreas in 20 (44.4%), lymph nodes in 20 (44.4%), lacrimal glands in 17 (37.8%), parotid glands in 14 (31.3%), biliary tract in 14 (31.1%), paranasal sinus in 13 (28.9%) and kidney in 11 (24.4%). Phenotyping classification and other clinical features are given in Table 1.

**Table 1:** Clinical characteristics of 45 IgG4-related disease patients.

Male, n (%)	24 (53.3)
Age at recruitment, mean ± SD	49.4 ± 15.3
Age at diagnosis, mean ± SD	47.3 ± 15.4
Delay in diagnosis, months, median (range)	19 (2–267)
Pancreato-hepato-biliary phenotype, n (%)	8 (17.7)
Retroperitoneum and aorta phenotype, n (%)	6 (13.3)
Head and neck-limited phenotype, n (%)	14 (31.1)
Mikulicz and systemic phenotype, n (%)	16 (35.5)
Unclassifiable phenotype, n (%)	1 (2.2)
Proliferative phenotype, n (%)	36 (80)
Fibrotic phenotype, n (%)	9 (20)
No. of organs involved (accrual), median (range)	4 (1–12)
No. of organs involved (at recruitment), median (range)	1 (0–10)
IgG4-RD RI at recruitment, median (range)	4 (0–24)
Active disease, n (%)	25 (55.5)
De novo active disease, n (%)	14 (56)
Relapsing disease, n (%)	9 (36)
Persistent active disease, n (%)	2 (8)
Damage, n (%)	35 (71.1)
Immunosuppressive treatment, n (%)	25 (55.5)
Glucocorticoids, n (%)	15 (33.3)
Immunosuppresors, n (%)	18 (40)
Azathioprine, n (%)	15 (33.3)
Mycofenolate mofetil, n (%)	3 (6.7)

*IgG4-RD RI* IgG4-related disease responder index.

IgG4 serum levels were elevated in 30 out of 44 (68.2%) patients ever tested during their disease course. At recruitment, serum IgG1 levels were elevated in 17 (37.7%) patients and serum IgG4 levels in 16 (35.5%), 10 with active IgG4-RD. Thirty five (77.7%) patients had biopsy proven disease, of which 26 had IgG4 + plasma cell count available.

Twenty five (55.5%) patients were under immunosuppressive treatment at recruitment. Type of immunosuppressive treatment is given in Table 1.

### sFLC assessment in patients with IgG4-RD

Twenty nine (64.4%) IgG4-RD patients had high κ sFLC levels with a median of 24.37 mg/L (1.61–287.96), 13 (28.9%) had high λ sFLC levels with a median of 19.19 mg/L (4.57–130.46) and 13 (28.9%) had an elevated κ/λ ratio with a median of 1.33 (0.35–3.92). Only one patient with inactive IgG4-RD had diminished κ and λ sFLC levels. There were no IgG4-RD patients with low κ/λ ratio.

**Figure 1:**
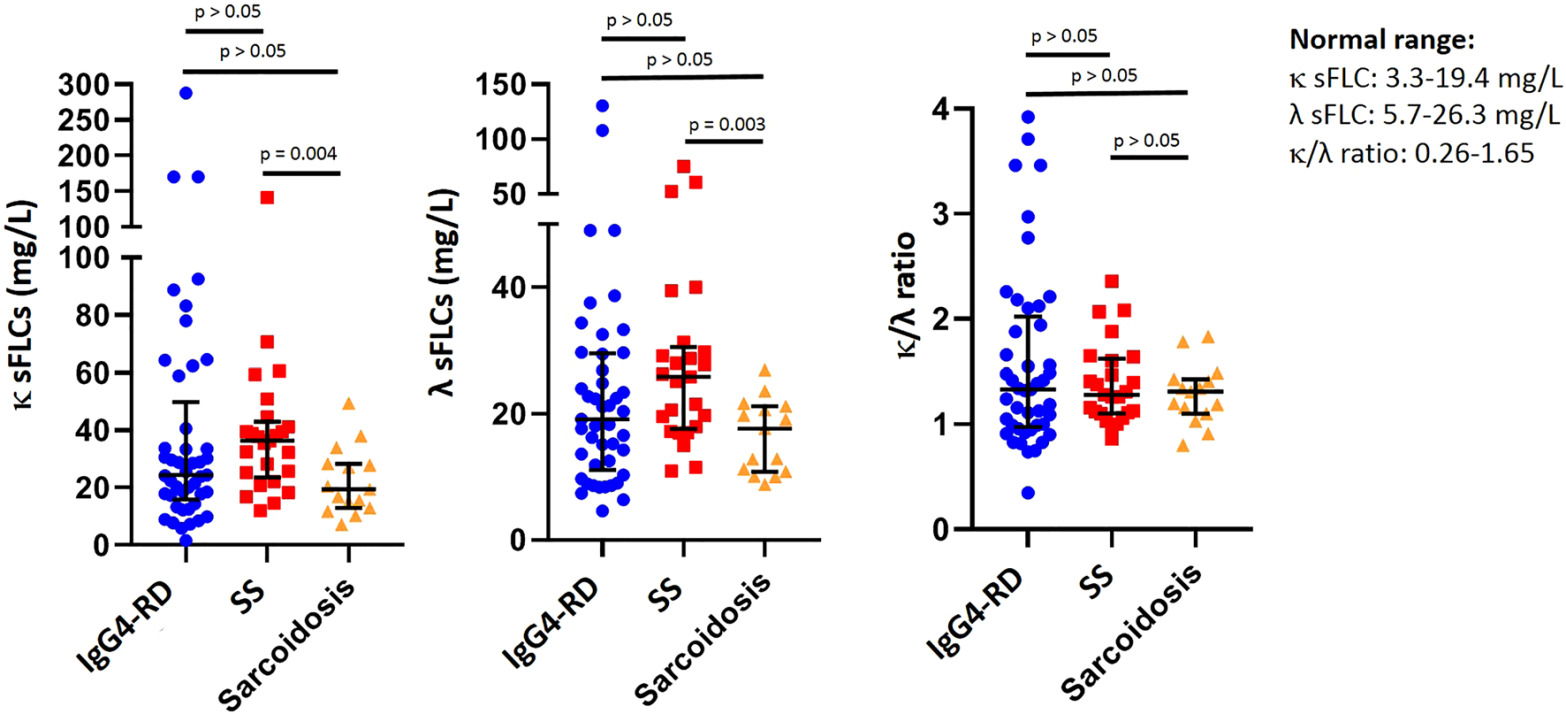
Levels of κ and λ serum free light chains and the κ/λ ratio were not different among IgG4-related disease (n = 45), Sjögren’s syndrome (n = 25) and sarcoidosis (n = 15) patients (median with interquantile range). Statistics performed by Kruskal–Wallis test. *sFLC* serum free light chains, *IgG4-RD* IgG4-related disease, *SS* Sjögren’s syndrome.

There were no differences in the proportion of IgG4-RD patients with high κ sFLC (29 [64.4%] vs. 21 [84%] vs. 8 [53.3%], p = 0.08) and elevated κ/λ ratio (13 [28.9%] vs. 4 [16%] vs. 2 [13.3%], p = 0.30) when compared with SS and sarcoidosis patients. On the contrary, patients with IgG4-RD had less frequently high levels of λ sFLC than SS patients and more frequently than sarcoidosis patients (13 [28.9%] vs.11 [44%] vs. 1 [6.7%], p = 0.04). Furthermore, the serum levels of κ sFLC, λ sFLC and κ/λ ratio were not significantly different among IgG4-RD patients and control groups (Fig. 1).

### sFLC in clinical phenotypes of IgG4-RD

**Figure 2:**
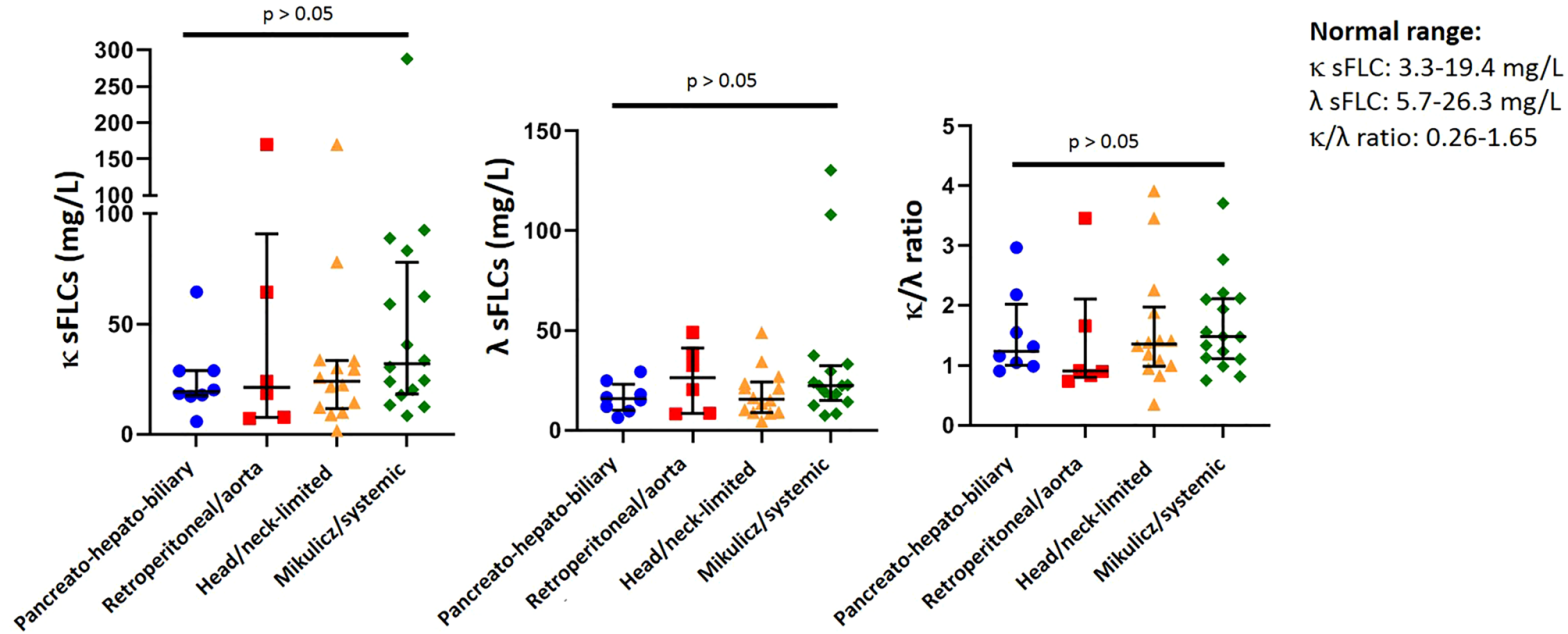
Levels of κ and λ serum free light chains and the κ/λ ratio were not different among pancreato-hepato-biliary (n = 8), retroperitoneum and aorta (n = 6), head and neck-limited (n = 14) and Mikulicz and systemic (n = 16) phenotypes (median with interquantile range). Statistics performed by Kruskal–Wallis test. *sFLC* serum free light chains.

There were no differences in the proportion of patients with high κ sFLC (4 [50%] vs. 3 [50%] vs. 9 [64.3%] vs. 12 (75%), p = 0.56), λ sFLC (1 [12.5%] vs. 3 [50%] vs. 3 [21.4%] vs. 5 (31.3%), p = 0.42) and κ/λ ratio (2 [25%] vs. 2 [33.3%] vs. 4 [28.6%] vs. 5 (31.3%), p = 0.98) among pancreato-hepato-biliary, retroperitoneum and aorta, head and neck-limited and Mikulicz and systemic phenotypes of IgG4-RD. Levels of κ sFLC, λ sFLC and κ/λ ratio were not significantly different among the four IgG4-RD clinical phenotypes (Fig. 2). The only patient with unclassifiable phenotype (pericardium limited involvement) was not included in this analysis.

**Figure 3:**
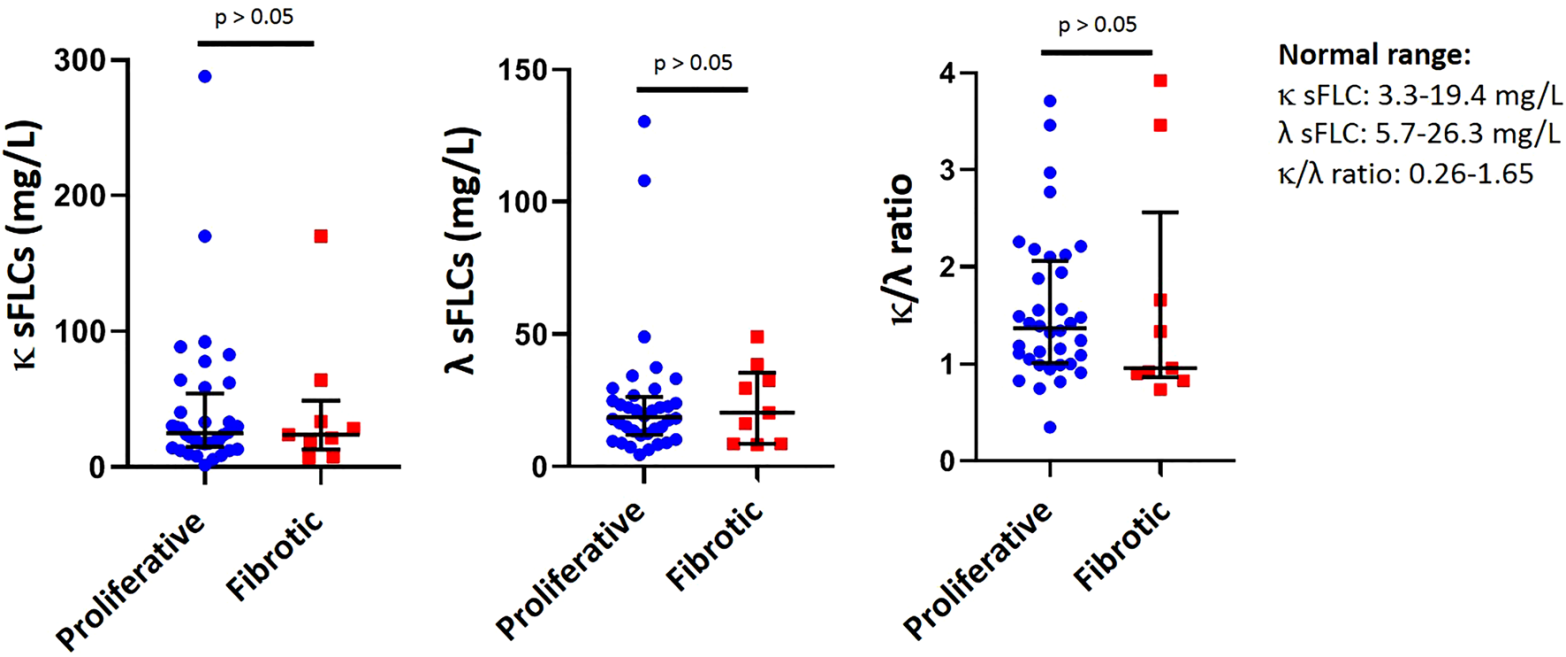
Levels of κ and λ serum free light chains and the κ/λ ratio were not different between proliferative (n = 36) and fibrotic (n = 9) phenotypes (median with interquantile range). Statistics performed by Mann–Whitney U test. *sFLC* serum free light chains.

There were no differences in the proportion of patients with high κ sFLC (23 [63.9%] vs. 6 [66.7%] p = 0.87), λ sFLC (9 [25%] vs. 4 [44.4%], p = 0.25) and κ/λ ratio (10 [27.8%] vs. 3 [33.3%], p = 0.74) between proliferative and fibrotic phenotypes of IgG4-RD. Levels of κ sFLC, λ sFLC and κ/λ ratio were not significantly different among these clinical phenotypes (Fig. 3).

### Relationship of sFLC and organ involvement in IgG4-RD

**Figure 4:**
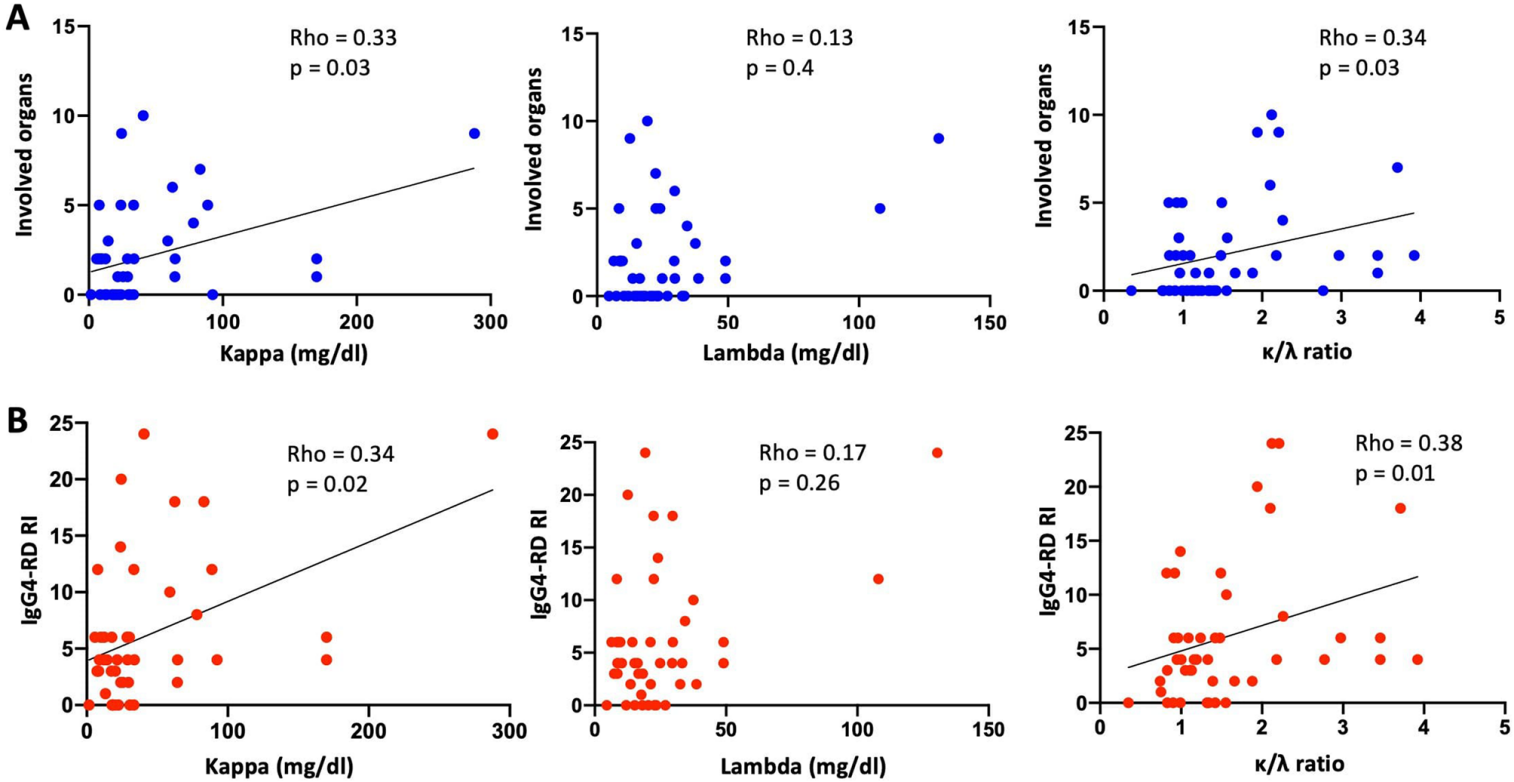
Relationship between κ and λ serum free light chains and the κ/λ ratio and clinical parameters in the IgG4-related disease patients (n = 45). (**A**) Correlation between the number of involved organs and the levels of κ and λ serum free light chains and the κ/λ ratio. (**B**) Correlation between the IgG4-RD RI and the levels of κ and λ serum free light chains and the κ/λ ratio. All correlations were determined using Spearman’s test. *IgG4-RD RI* IgG4-related disease responder index.

We observed a significant positive correlation between the number of involved organs at recruitment and κ sFLC (rho = 0.33, p = 0.03) and the κ/λ ratio (rho = 0.34, p = 0.02), but not with λ sFLC (rho = 0.13, p = 0.40) (Fig. 4). A positive correlation with the IgG4-RD RI score was also observed with κ sFLC (rho = 0.34, p = 0.02) and the κ/λ ratio (rho = 0.38, p = 0.01), but not with λ sFLC (rho = 0.17, p = 0.26) (Fig. 4).

**Figure 5:**
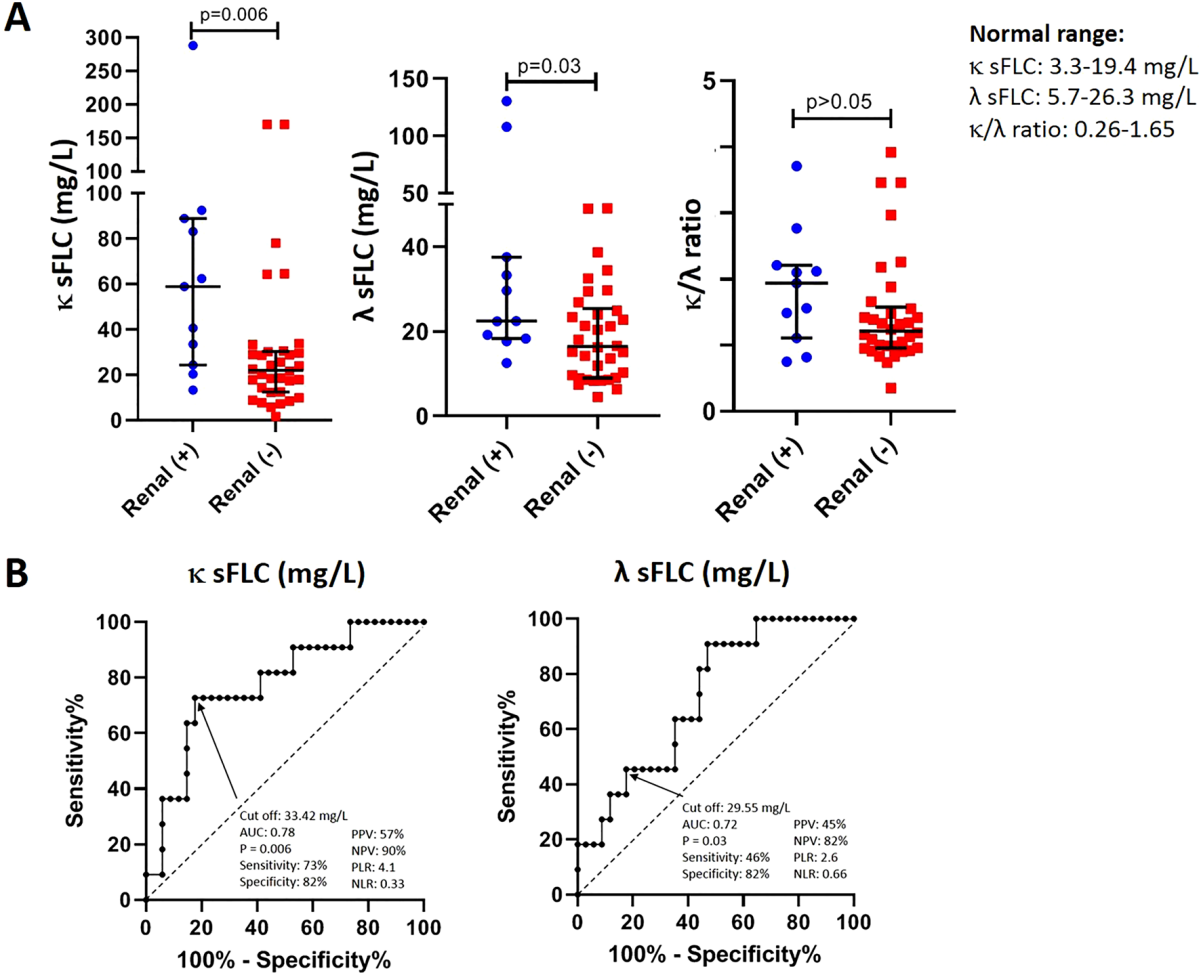
(**A**) Levels of κ and λ serum free light chains were different between IgG4-related disease patients with (n = 11) compare to those without (n = 34) renal involvement (median with interquantile range). Statistics performed by Mann–Whitney U test. (**B**) The receiver operating characteristic curve for κ and λ serum free light chains distinguished patients with renal involvement from patients without. *sFLC* serum free light chains, *PPV* positive predictive value, *NPV* negative predictive value, *PLR* positive likelihood ratio, *NLR* negative likelihood ratio.

Patients with renal involvement (n = 11 [24.4%]) had higher levels of κ sFLC (58.9 mg/L [13.32–287.96] vs. 22.03 mg/L [1.61–170.66], p = 0.006) and λ sFLC (22.48 mg/L [12.55–130.46] vs. 16.43 mg/L [4.57–49.50], p = 0.03) compared to patients without renal involvement (Fig. 5); on the contrary, there were no differences in the levels of serum IgG1 (852 mg/dL [199–2662] vs. 745 mg/dL [252–1300], p = 0.19) and IgG4 115 mg/dL [7–1088] vs. 68 mg/dL [8–192], p = 0.06) between both groups.

The AUC for κ sFLC and λ sFLC, for predicting renal involvement, with a cut-off value of 33.42 mg/L and 29.55 mg/, was 0.78 and 0.72, respectively (Fig. 5).

Only 2 patients with renal involvement had mild kidney dysfunction (eGFR 60–89 mL/min/1.73 m^2^) and the rest had normal kidney function; eight had active disease. At the linear regression analysis, eGFR did not correlate with κ (β = − 0.03, p = 0.92) and λ sFLC levels (β = 0.05, p = 0.74) nor the κ/λ ratio (β = − 0.008, p = 0.17).

Patients with lymph node involvement (n = 20, 44.4%) had higher levels of κ sFLC (31.94 mg/L [8.40–287.96] vs. 21.63 mg/L [1.61–170.06], p = 0.026) and a trend for higher levels λ sFLC (21.89 mg/L [7.40–130.46] vs. 16.26 mg/L [4.57–49.06], p = 0.058) compare to patients without lymph node involvement. We did not find any association with other organs.

### Relationship of sFLC with activity and damage in IgG4-RD

Twenty-five of the IgG4-RD patients had active disease, of whom 14 (56%) had de novo, nine (36%) relapsing and two (8%) persistent active disease.

**Figure 6:**
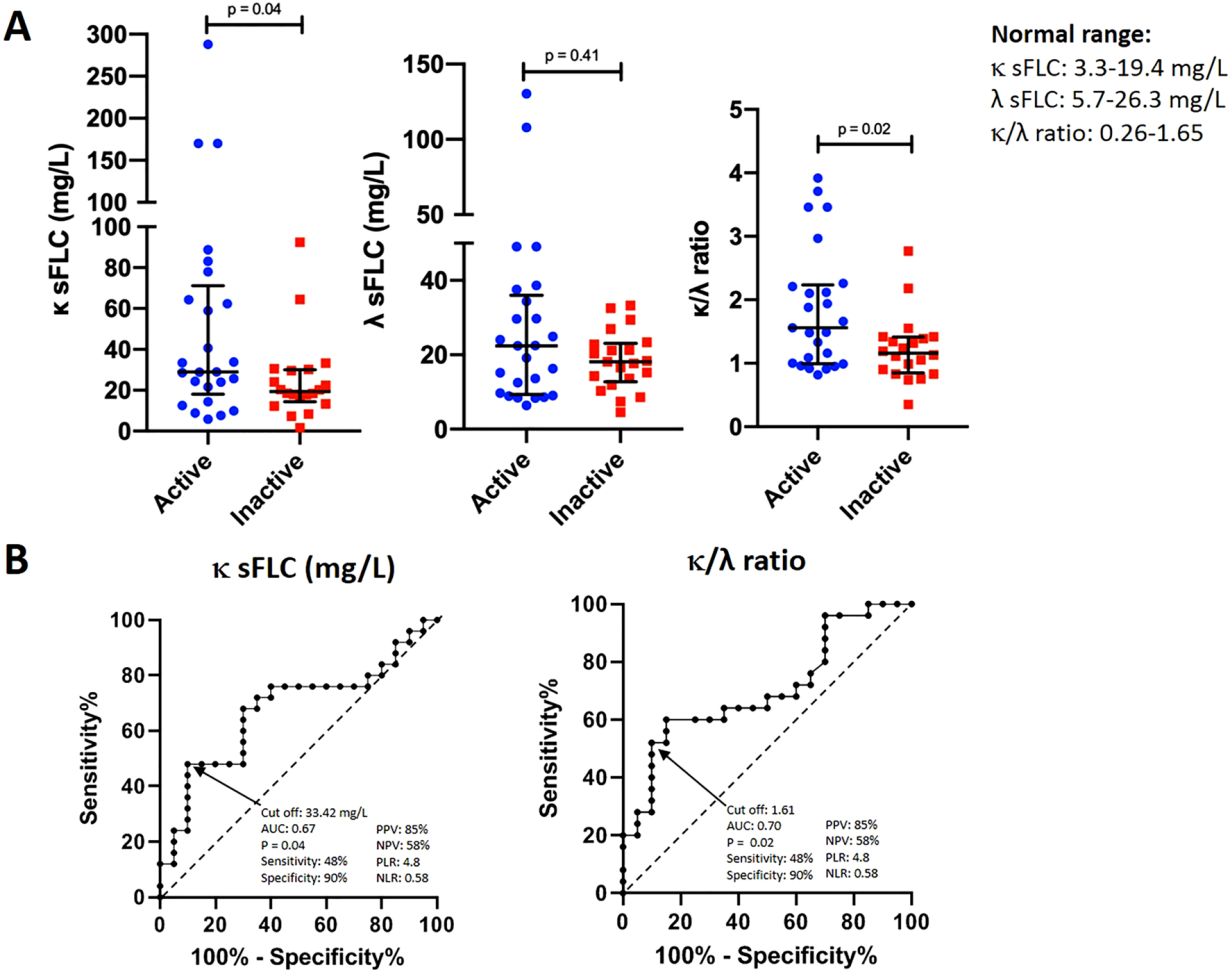
(**A**) Levels of κ serum free light chains and the κ/λ ratio were different between active (n = 25) compare to inactive (n = 20) IgG4-related disease patients (median with interquantile range). Statistics performed by Mann–Whitney U test. (**B**) The receiver operating characteristic curve for κ serum free light chains and the κ/λ ratio distinguished patients with active from inactive disease. *sFLC* serum free light chains, *PPV* positive predictive value, *NPV* negative predictive value, *PLR* positive likelihood ratio, *NLR* negative likelihood ratio.

Patients with active IgG4-RD had more frequently high κ sFLC compared to patients with inactive IgG4-RD, although without reaching a statistical significance (19 [76%] vs. 10 [50%], p = 0.07); whereas the proportion of patients with high λ sFLC was similar (9 [36%] vs. 4 [20%], p = 0.23). Patients with active IgG4-RD had more frequently an elevated κ/λ ratio than patients with inactive disease (12 [48%] vs. 2 [10%], p = 0.006). The concentrations of κ sFLC (28.95 mg/L [5.83–287.96] vs. 19.37 mg/L [1.61–92.45], p = 0.04) and the κ/λ ratio (1.56 [0.82–3.92] vs. 1.16 [0.35–2.77], p = 0.02) were higher in active than in inactive IgG4-RD (Fig. 6); on the contrary, there were no differences in the levels of serum IgG1 (852 mg/dL [394–2433] vs. 745 mg/dL [199–2662], p = 0.16) and IgG4 (72.5 mg/dL [7–1088] vs. 114 mg/dL [36–1050], p = 0.10) between both groups. Notably, half of the 12 active IgG4-RD patients with a high κ/λ ratio had normal serum IgG4 levels, four of them had de novo active disease and two relapsing disease.

The AUC for κ sFLC and κ/λ ratio, for predicting active IgG4-RD, with a cut-off value of 33.42 mg/L and 1.61, were 0.67 and 0.70, respectively (Fig. 6).

In a sensitivity analysis including only the 14 patients with de novo active disease, we also found a higher level of κ sFLC (33.63 mg/L [57.72–170.06] vs. 19.37 mg/L [1.61–92.45], p = 0.014) and higher κ/λ ratio (1.99 [0.92–3.92] vs. 1.16 [0.35–2.77], p = 0.017). The AUC for κ sFLC and κ/λ ratio for discriminating de novo active from inactive disease with the same cut-off values (33.42 mg/L and 1.61) were 0.75 and 0.74, respectively. On the contrary, when including only the nine patients with relapsing disease, there were no differences compare to the inactive group (data not shown).

IgG4-RD patients with damage at the time of recruitment did not have differences in sFLC levels compare to patients without damage (data not shown).

### Relationship of sFLC and serum IgG1 and IgG4 levels and histopathology

**Figure 7:**
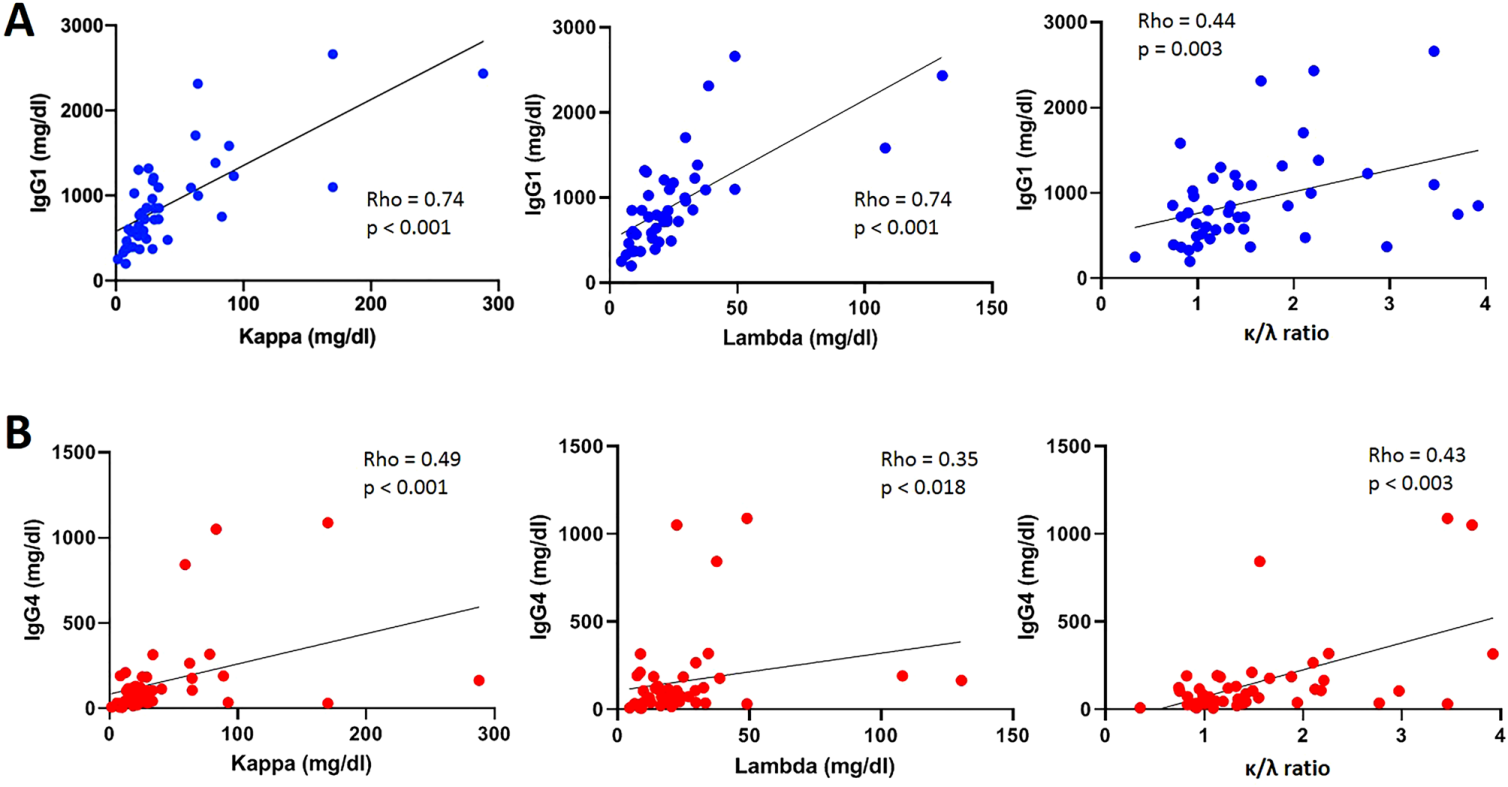
Relationship between κ and λ serum free light chains and the κ/λ ratio and serological parameters in the IgG4-RD patients (n = 45). (**A**) Correlation between serum IgG1 levels and the levels of κ and λ serum free light chains and the κ/λ ratio. (**B**) Correlation between serum IgG4 and the levels of κ and λ serum free light chains and the κ/λ ratio. All correlations were determined using Spearman’s test.

There was a significant positive correlation between serum IgG1 levels and κ sFLC (rho = 0.74, p < 0.001), λ sFLC (rho = 0.74, p < 0.001) and the κ/λ ratio (rho = 0.44, p = 0.003) (Fig. 7). Serum IgG4 levels correlated as well with κ sFLC (rho = 0.49, p < 0.001), λ sFLC (rho = 0.35, p = 0.018) and the κ/λ ratio (rho = 0.43, p = 0.003) (Fig. 7).

Patients with biopsy proven disease (n = 35) have no differences in the prevalence κ sFLC, λ sFLC and the κ/λ ratio and their levels compare to patients without biopsy proven disease (n = 10) (data not shown). The number of IgG4 + plasma cells in tissue did not correlate with κ sFLC (rho = 0.23, p = 0.25) λ sFLC (rho = 0.16, p = 0.41) nor the κ/λ ratio (rho = 0.28, p = 0.16).

### sFLC in IgG4-RD patients under immunosuppressive treatment

There were no differences in the prevalence and levels of κ sFLC, λ sFLC and the κ/λ ratio between patients with and without immunosuppressive treatment (data not shown).

In a sensitivity analysis including the 14 patients with active untreated IgG4-RD, an elevated κ/λ ratio remained more frequent compare to SS and sarcoidosis patients (7 [50%] vs. 4 [16%] vs. 2 (13.3), p = 0.04).

## Discussion

In this study we demonstrated that a substantial proportion of patients with IgG4-RD had high levels of sFLC, and although their assessment may not be useful for distinguishing from other conditions, it may be valuable as a biomarker of disease activity and multiorgan and renal involvement. Additionally, a high κ/λ ratio may be valuable for the same purposes.

To date, only one study have assessed sFLC levels in patients with IgG4-RD. Grados et al. determined sFLC levels in 16 French patients with IgG4-RD and found that they were higher compared to a group of healthy subjects; furthermore, they found that 87% and 53% of the patients had elevated κ and λ sFLC respectively. However, due to the small sample size they were not able to assess the relationship of sFLC with clinical characteristics and disease activity^20^. In our study, we found that 64.4% and 28.9% of our cohort had elevated κ and λ sFLC respectively, which is lower than the frequencies reported by Grados et al. These differences may be explained by the fact that in the French study patients with severe kidney impairment were also included and it is well established that sFLC correlate negatively with the degree of renal insufficiency^21^.

The increase levels of sFLC in patients with IgG4-RD is undoubtedly related to the increase synthesis of immunoglobulins by the B cell lineage which also could explain why we found a positive correlation between sFLC and IgG1 and IgG4 levels. This phenomenon is well described in systemic autoimmune diseases in which B cell over-activation is a component of the pathogenesis of the disease such as SS and SLE^4,5^. It was noteworthy that a considerable proportion of our IgG4-RD cohort (28.9%) had an abnormal κ/λ ratio, in contrast to the lower frequencies found in SS and SLE^4,22^. Skewing of the κ/λ ratio reflects the excess production of one immunoglobulin light chain over the other and is considered a surrogate laboratory parameter indicating clonality in plasmaproliferative or lymphoproliferative disorders^2^. This pattern of κ-restricted pseudoclonality has been previously reported in IgG4-RD and in settings where there is polyclonal IgG4 excess. Grados et al. found an elevated κ/λ ratio of sFLC in 44% of 16 patients with IgG4-RD^20^. Furthermore, two studies have demonstrated that sera from patients with increased serum IgG4 levels evaluated by immunosubstraction, including patients with IgG4-RD, may have a monotypic appearance with a κ-restricted pseudoclonal pattern^23,24^. The observed κ-restricted pseudoclonality may be attributable to the intrinsic characteristics of the IgG4 subclass itself. The ratio of κ- to λ-expressing IgG, commonly 2 to 1, depends on the IgG subclass. IgG1, for example, has a κ/λ ratio of 2.4 to 1, whereas IgG4 has a κ/λ ratio of about 8 to 1 with a heavily skewing towards the κ light chain^24^. This κ-restricted pseudoclonality seen by immunosubstraction and by measurement of sFLC, together with the polyclonal IgG4 serum protein electrophoresis pattern that may mimic a monoclonal gammopathy^23,24^, may lead to a presumed diagnosis of clonality. While some cases of IgG4-related ophthalmic disease with κ light chain restriction evaluated by in-situ hybridization have been reported^25,26^, if tissue specimens from IgG4-RD patients had the same immunoglobulin light chain pseudo-restriction remains to be determined.

There is an unmet need regarding biomarkers in IgG4-RD. Up to date, only serum IgG4 and complement levels have a role in diagnosis and/or classification of the disease^20,27^. Furthermore, whereas serum IgG4 levels are a valuable tool to disclose multi-systemic disease and risk of relapse, complement levels may predict renal involvement^16,28^. However, no single serological feature is by itself specific enough for the diagnosis of IgG4-RD. Data on biomarkers to differentiate IgG4-RD from other conditions have been conflicting. For instance, Akiyama et al. described that serum soluble IL-2 receptor was not useful for the differential diagnosis between IgG4-RD and SS^17^. On the contrary, a recent study by Umeda et al. found that serum thymus and activation-regulated chemokine could discriminate between IgG4-RD and SS^29^. In our study we did not find differences in sFLC concentrations among IgG4-RD group and both control groups, however, up to half of the patients with active IgG4-RD had an elevated κ/λ ratio.

As has been described for other systemic autoimmune diseases such as SLE, SS, RA and SSc^4–7^, measurement of sFLC may be a useful biomarker to assess systemic activity in IgG4-RD, as demonstrated by our findings of higher levels of κ sFLC and κ/λ ratio in patients with active vs. inactive IgG4-RD. Interestingly, only two patients with inactive disease had an elevated κ/λ ratio. Furthermore, half of the patients with an elevated κ/λ ratio had normal serum IgG4 levels, thus, an elevated κ/λ ratio could point to active disease even in those patients with normal serum IgG4. Interestingly, most of the active patients with an elevated κ/λ ratio had de novo active disease.

In certain clinical scenarios after treatment of IgG4-RD, it may be difficult to assess whether a clinical or imaging finding is a reflection of ongoing organ inflammation and activity or permanent scarring and damage; this is especially true for clinical manifestations of the fibrotic phenotype such as retroperitoneal fibrosis and some cases of orbital pseudotumor. In other settings, biochemical findings reflecting permanent damage such as persistent elevation of alkaline phosphatase or urine protein may lead to the false reassurance of ongoing biliary tract and kidney activity followed by unnecessary treatment. Thus, a biomarker for disease activity in IgG4-RD may be particularly beneficial in these situations. The ROC curve generated by the results in the present study showed that the cut-off value of 33.42 mg/L for κ sFLC and 1.61 for the κ/λ ratio may discriminate between active and inactive IgG4-RD with a high specificity and positive predictive value, although with a low sensitivity. Interestingly, the AUC for de novo active disease had a better performance, while this was not true for relapsing disease. The latter finding may be due to the smaller number of patients with relapsing disease compare to de novo active disease included in our study.

In agreement with the aforementioned findings, we found a positive correlation between the number of organs involved and the IgG4-RD RI with both the κ sFLC and the κ/λ ratio. Furthermore, we observed higher levels of sFLC in patients with lymph node involvement. As suggested by Grados et al. these findings may correlate with the volume of the polyclonal “tumoral” infiltrate of IgG4-RD^20^, as has been reported for multiple myeloma and AL amyloidosis where sFLC levels correlate with bone marrow infiltration by plasma cells and the number of organs involved by amyloid, respectively^30,31^.

Nowadays, classifying patients with IgG4-RD in different phenotypic groups is appealing^11,23^. Differences in laboratory parameters among different subsets is well stablished in terms of IgG, IgG4, IgE, eosinophils and complement levels. It was tempting to hypothesize that patients belonging to the Mikulicz and systemic and the proliferative phenotype would display higher sFLC levels, as these phenotypes more frequently present with polyclonal hypergammagobulinemia; however, we did not find differences in sFLC levels among the distinct IgG4-RD phenotypes.

Interestingly, we observed that concentrations of both κ and λ sFLC were higher in IgG4-RD patients with kidney involvement, although this observation was not driven by a low eGFR. The ROC curves showed that the cut-off value of 33.42 mg/L for κ sFLC had an acceptable sensitivity and specificity, with a negative predictive value of 90%, rendering κ sFLC as an excellent tool to exclude kidney involvement.

Our study is not exempt of limitations. First, the study had a cross sectional design and thus we did not measure sFLC prospectively to assess their utility to predict relapses and treatment response. Second, serum and urine electrophoresis and immunofixation was not routinely done in our patients to exclude a monoclonal plasma cell disorder, nevertheless, we have no doubt about the certainty of IgG4-RD diagnosis. Third, although some patients were under immunosuppressive therapy at the time of recruitment, we did not find differences in sFLC levels among patients with vs. without such therapy. Finally, longitudinally studies are needed to better define if sFLC levels are sensitive to change under immunosuppressive therapy and for predicting relapsing disease.

Some strengths also need to be mentioned. First, this is the first study to evaluate the usefulness of sFLC in cohort of IgG4-RD according to the clinical phenotype and activity status. Second, we included a control group with other systemic autoimmune diseases that commonly mimic IgG4-RD in order to assess their value as a complementary diagnostic tool. Nevertheless, the performance of sFLC might be further evaluated in other IgG4-RD mimickers such as malignancies or ANCA-associated vasculitides.

## Conclusions

In summary, a significant proportion of patients with IgG4-RD may present with high levels of sFLC and, even though their assessment may not be useful for distinguishing from SS and sarcoidosis. Measurement of sFLC may be useful as a biomarker of disease activity and multiorgan and renal involvement. In particular, a high κ/λ ratio may identify patients with active disease even in those with normal IgG4 levels.

## Methods

### Patients

This was a cross-sectional study. We included consecutive patients with a diagnosis of IgG4-related disease according to the Comprehensive Diagnostic Criteria for IgG4-related disease^32^ who attended a referral center in Mexico City from August 2018 to November 2019. We excluded patients with chronic kidney disease (estimated glomerular filtration rate (eGFR) < 60 mL/min/1.73 m^2^ for ≥ 3 months), concomitant diagnosis of another systemic autoimmune/inflammatory disease, active infection or malignancy, including monoclonal gammopathies. As a control group, we included patients with SS according to the 2016 American College of Rheumatology/European League Against Rheumatism classification criteria for SS^33^ and patients with sarcoidosis according to the American Thoracic Society/European Respiratory Society/World Association of Sarcoidosis and other Granulomatous Disorders diagnostic criteria^34^. We obtained approval from the Institutional Review Board (Comité de Ética en Investigación del Instituto Nacional de Ciencias Médicas y Nutrición Salvador Zubirán, IRE-2549-18-20-1) and the study complies with the Declaration of Helsinki. Patients and controls gave written informed consent.

We retrospectively collected patient information such age at diagnosis, number of organs ever involved, IgG4 serum levels at diagnosis and/or during the disease course, biopsy availability and the use of glucocorticoids and immunosuppressive therapy.

Patients were classified according to clinical phenotypes as described by Wallace et al. in pancreato-hepato-biliary, retroperitoneum and aorta, head and neck-limited and Mikulicz and systemic phenotypes^11^; patients with only mesentery or mediastinal involvement were included in the retroperitoneum and aorta phenotype; and patients who could not be fitted in one of these clinical phenotypes were termed “unclassifiable” phenotype. Patients were also classified according to the clinical phenotypes described by Zhang et al. in proliferative and fibrotic phenotypes^35^. We assessed the number of involved organs and the IgG4-RD Responder Index (IgG4-RD RI) at recruitment^36^.

We defined active disease as the presence of clinical signs and symptoms, laboratory abnormalities or unequivocal radiological findings attributable to IgG4-RD. Patients with active disease included those with de novo active disease (first episode of active IgG4-RD), relapsing disease (active disease after a period of inactive IgG4-RD) and persistent active disease (active disease despite immunosuppressive treatment). We defined inactive disease as the absence of clinical signs and symptoms, laboratory abnormalities and new unequivocal radiological findings attributable to IgG4-RD. Damage was defined as irreversible organ dysfunction or failure caused by IgG4-RD or as a consequence of surgical procedures performed to diagnose or treat IgG4-RD^36^.

### sFLC assessment

Blood samples from patients and controls were collected. Serum samples were extracted and frozen at − 20 °C until processing. sFLC levels were measured in the same laboratory using latex-enhanced immunoassay (Freelite, The Binding Site, UK) using turbidimetry (SPA_PLUS_). The immunoassay consisted of two separate measurements, one for free κ and one for free λ and then the automatic calculation of the κ/λ ratio. Normal values were set according to the manufacturer’s recommendations as follows: normal free κ range: 3.3–19.4 mg/L; normal free λ range: 5.7–26.3 mg/L; and normal κ/λ ratio range: 0.26–1.65^37^. In patients with IgG4-RD, serum IgG1 and IgG4 levels were determined with turbidimetry analyzer (SPA_PLUS_, Freelite, The Binding Site, UK) and normal values were also set according to the manufacturer’s recommendations as follows: IgG1 normal range: 382.4–928.6 mg/dL; IgG4 normal range: 3.9–86.4 mg/dL. eGFR was calculated with the Chronic Kidney Disease Epidemiology Collaboration equation at the time of recruitment.

### Statistical analysis

We used descriptive statistics. Dichotomous variables were expressed as absolute frequencies and continuous variables as means and standard deviations (SD) or medians and minimum and maximum range as appropriate. Comparison between means was made with Student’s T-test and between medians with Mann–Whitney U test. Categorical variables were analyzed with Chi square test or Fisher exact test as appropriate. We used Kruskal–Wallis test for comparison among multiple groups. Correlations among variables were evaluated using Spearman’s test.

We estimated the sensitivity, specificity, positive predictive value and negative predictive value, as area under the curve (AUC) for predicting active disease and kidney involvement. Finally, we conducted a linear regression analysis to elicit GFR as a predictor of sFLC levels. A two-tailed P < 0.05 was considered statistically significant. All analyses were performed using the SPSS 20.0 and GraphPad Prism 8.3.0 for Windows 20.0.

## Data Availability

The datasets used during the current study are available from the corresponding author on reasonable request.
