# Author Correction: Serum immunoglobulin free light chains and their association with clinical phenotypes, serology and activity in patients with IgG4‑related disease

**DOI:** 10.1038/s41598-021-91558-9

**Published:** 2021-06-01

**Authors:** Eduardo Martín‑Nares, Vanessa Saavedra‑González, Reynerio Fagundo‑Sierra, Blanca Estela Santinelli‑Núñez, Teresa Romero‑Maceda, Karla Calderón‑Vasquez, Gabriela Hernandez‑Molina

**Affiliations:** 1 Department of Immunology and Rheumatology, Instituto Nacional de Ciencias Médicas y Nutrición Salvador Zubirán, Vasco de Quiroga 15, Col. Belisario Dominguez Sección XVI, 14080 Mexico City, Mexico; 2 Central Laboratory, Instituto Nacional de Ciencias Médicas y Nutrición Salvador Zubirán, Mexico City, Mexico; 3 Clinical Laboratory, Tumor Markers Unit, Instituto Nacional de Cancerología, Mexico City, Mexico

Correction to: *Scientific Reports* 10.1038/s41598-021-81321-5, published online 19 January 2021

The original version of this Article contained errors in Figure 4, where panel (b) was duplicated from panel (a). As a result, the red value dots in panel (b) and the y-axis labels were displayed incorrectly. The original Figure 4 and accompanying legend appear below.

**Figure 4:**
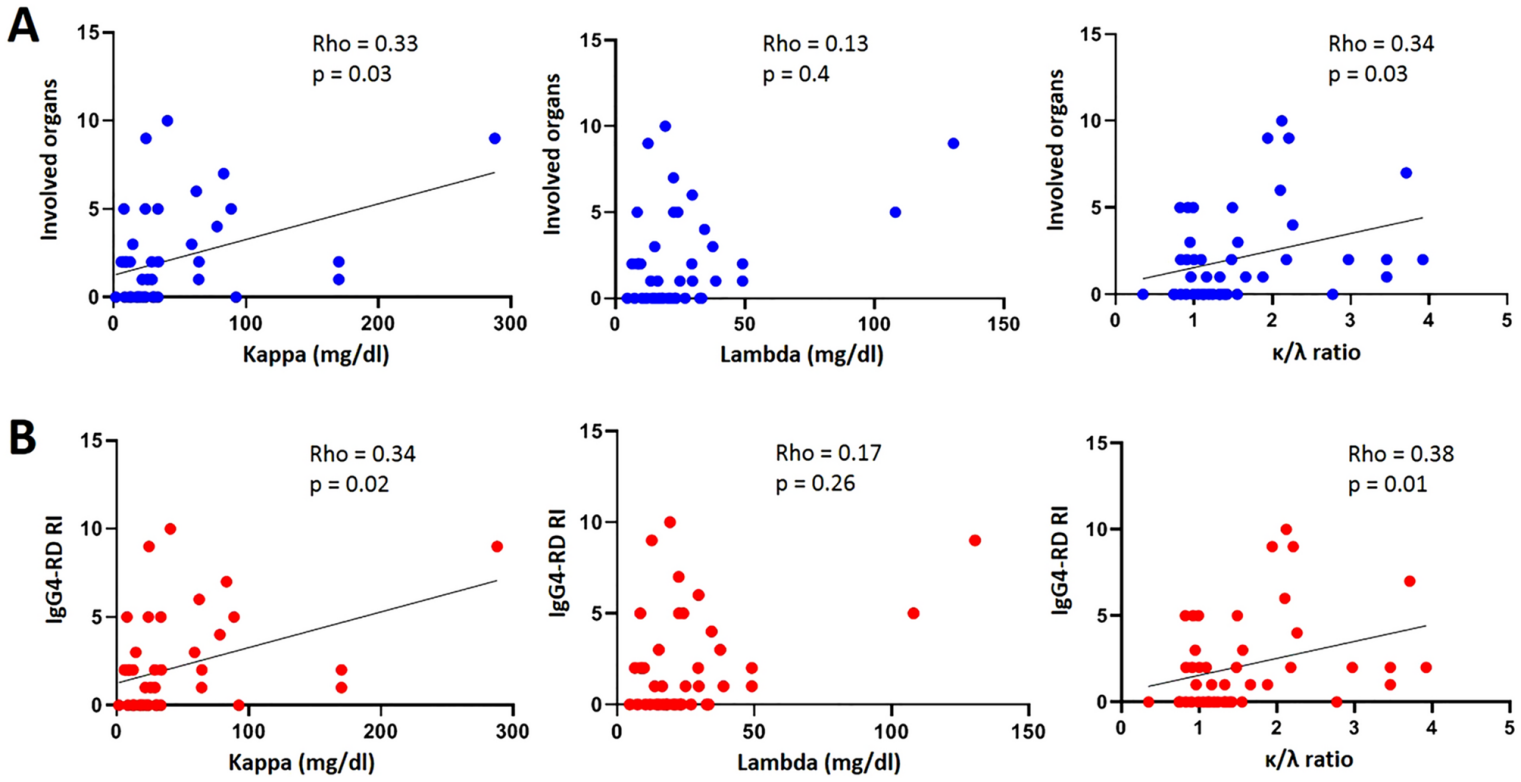
Relationship between κ and λ serum free light chains and the κ/λ ratio and clinical parameters in the IgG4-related disease patients (n = 45). (**A**) Correlation between the number of involved organs and the levels of κ and λ serum free light chains and the κ/λ ratio. (**B**) Correlation between the IgG4-RD RI and the levels of κ and λ serum free light chains and the κ/λ ratio. All correlations were determined using Spearman’s test. *IgG4-RD RI* IgG4-related disease responder index.

The original Article has been corrected.

